# Long-term smoking alters abundance of over half of the proteome in bronchoalveolar lavage cell in smokers with normal spirometry, with effects on molecular pathways associated with COPD

**DOI:** 10.1186/s12931-017-0695-6

**Published:** 2018-03-08

**Authors:** Mingxing Yang, Maxie Kohler, Tina Heyder, Helena Forsslund, Hilde K. Garberg, Reza Karimi, Johan Grunewald, Frode S. Berven, C. Magnus Sköld, Åsa M. Wheelock

**Affiliations:** 1 Department of Medicine Solna & Center for Molecular Medicine, Respiratory Medicine Unit, Lung Research Lab L4:01, Karolinska Institutet, 171 76 Stockholm, Sweden; 2 Department of Biomedicine, Proteomics Unit (PROBE), University of Bergen, Bergen, Norway

**Keywords:** Smoking, Inflammation, Proteomics, COPD, Bronchoalveolar lavage

## Abstract

**Background:**

Smoking represents a significant risk factor for many chronic inflammatory diseases, including chronic obstructive pulmonary disease (COPD).

**Methods:**

To identify dysregulation of specific proteins and pathways in bronchoalveolar lavage (BAL) cells associated with smoking, isobaric tags for relative and absolute quantitation (iTRAQ)-based shotgun proteomics analyses were performed on BAL cells from healthy never-smokers and smokers with normal lung function from the Karolinska COSMIC cohort. Multivariate statistical modeling, multivariate correlations with clinical data, and pathway enrichment analysis were performed.

**Results:**

Smoking exerted a significant impact on the BAL cell proteome, with more than 500 proteins representing 15 molecular pathways altered due to smoking. The majority of these alterations occurred in a gender-independent manner. The phagosomal- and leukocyte trans endothelial migration (LTM) pathways significantly correlated with FEV_1_/FVC as well as the percentage of CD8^+^ T-cells and CD8^+^CD69^+^ T-cells in smokers. The correlations to clinical parameters in healthy never-smokers were minor.

**Conclusion:**

The significant correlations of proteins in the phagosome- and LTM pathways with activated cytotoxic T-cells (CD69+) and the level of airway obstruction (FEV_1_/FVC) in smokers, both hallmarks of COPD, suggests that these two pathways may play a role in the molecular events preceding the development of COPD in susceptible smokers. Both pathways were found to be further dysregulated in COPD patients from the same cohort, thereby providing further support to this hypothesis. Given that not all smokers develop COPD in spite of decades of smoking, it is also plausible that some of the molecular pathways associated with response to smoking exert protective mechanisms to smoking-related pathologies in resilient individuals.

**Trial registration:**

ClinicalTrials.gov identifier NCT02627872; Retrospectively registered on December 9, 2015.

**Electronic supplementary material:**

The online version of this article (10.1186/s12931-017-0695-6) contains supplementary material, which is available to authorized users.

## Background

The number of habitual smokers is estimated to one billion worldwide [1]. The direct effect of tobacco smoking was responsible for the death of 6 million people in 2015, with 27% of them resulting from COPD [2]. Although one-third of long-term smokers develop COPD [3], the molecular mechanisms by which smoking-induced inflammation triggers the development of disease remain unclear.

Proteomic profiling of blood, urine and lung tissue from smokers has improved our understanding of the molecular impact of smoking [4–6]. Lung immune cells, especially alveolar macrophages, play a key role in the immune responses to smoking [7]. However, quantitative proteomics analysis of resident immune cells in the lung, particularly with respect to the inflammatory disorder in the response to smoking, are scarce. Our previous work revealed specific alterations of the bronchoalveolar lavage (BAL) cell proteome in current-smoker COPD patients [8]. However, in order to define molecular disease mechanisms occurring prior to disease manifestations, alterations of the BAL cell proteome in response to smoking needs to be defined.

The aim of this study was to investigate the impact of long-term habitual smoking on the BAL immune cell proteome. The proteomes of long-term smokers at elevated risk of developing COPD were contrasted to healthy never-smokers, with the specific emphasis on identifying alterations of molecular pathways occurring prior to the presentation of symptoms or decline of lung function associated with COPD diagnosis.

## Methods

The details of methods are provided in Supplemental materials.

### Study subjects and design

**Fig. 1:**
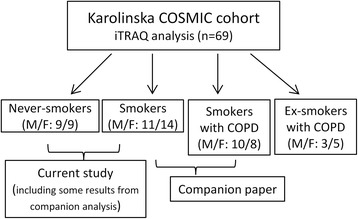
Flow chart of the study design outlining groups of the current paper as well as the companion manuscript [15]. A total of 69 subjects from the Karolinska COSMIC cohort, age- and gender- matched, were selected for iTRAQ proteomic investigations, including 18 healthy Never-smokers, 25 Smokers with normal lung function, 18 current smokers with COPD, and 8 ex-smokers with COPD. The current analysis consists of 17 Never-smokers (one male Never-smoker did not pass QC and was excluded from further analysis), 25 Smokers with normal lung function. This report focuses on the alterations in proteomes and pathways related to smokingwhereas the pathways related to pathogenesis of COPD are reported in a companion paper [15]

This study was carried out on subjects from the Karolinska COSMIC cohort [8–13], a three-group cross-sectional study including age- (45–65 years) and sex-matched groups of healthy never-smokers (hereafter referred to as Never-smokers), smokers with normal lung function (hereafter referred to as Smokers; >10 pack years; >10 cigarettes/day the past 6 months), and smokers with COPD (GOLD stage I-II/A-B, FEV_1_ > 50% of predicted and FEV_1_/FVC < 0.7). Sixty-nine subjects were selected for proteome analyses (Fig. 1). The current segment included Never-smokers (*n* = 17) and Smokers (*n* = 25). Acute effects of smoking were excluded by requesting that participants to refrain from smoking at least 8 h prior to biospecimen collection, as confirmed by exhaled carbon monoxide monitoring [14]. Results from the Smokers compared to the COPD group are presented in the companion paper [15]. All subjects underwent extensive clinical examinations, including computed tomography [10, 13] and spirometry (Table 1, Additional file 1: Table S1). BAL cells were collected during bronchoscopy as previously described [16]. Clinical characteristics are summarized in Table 1. The study was approved by the Stockholm regional ethical board (Case no. 2006/959–31/1), and written informed consent was obtained from all subjects.

**Table 1:** The clinical characteristics of subjects

	Never-smoker (*n* = 17)	Smoker (n = 25)
Male/female	8/9	11/14
Age	56.2 ± 7.3	56.2 ± 5.7
BMI	26.4 ± 5.5	25.2 ± 2.9
Pack years	0	38.1 ± 13.5
Cig/day during past 6 months	0	17.1 ± 6.3
FEV_1_ [%] post-bronchodilator	118 ± 15.8	107 ± 12.8*
FEV_1_/FVC [%] post-bronchodilator	80.5 ± 6.4	77.7 ± 5.1
BAL macrophages [%]	90.2 ± 3.8	95.9 ± 2.4***
BAL lymphocytes [%]^a^	6.6(3, 15.4)	2(1,7.4)
BAL neutrophils [%]^a^	1.3 (0.6, 4.4)	0.6(0, 3.6)*
BAL eosinophils [%]^a^	0(0, 0.6)	0(0, 1.4)
BAL basophils [%]^a^	0(0, 0)	0(0, 0.4)
BAL mast cells [per 10 visual fields]	2(0, 5)	3(0, 13)

Data is expressed as mean ± standard deviation and tested by using t-test.^a^, skew data, presented as median(range) and tested by Mann-Whitney U test; *, *p* < 0.05; ***, *p* < 0.001, compared with Never-smokers

### Proteomic analyses

Trypsinized protein extracted from 1.5 × 10^6^ BAL cells, were used for 4-plexed iTRAQ labeling (AB SCIEX). The 114 tag was dedicated to a pooled reference sample in all sets, other samples were randomized and labeled with the 115, 116 and 117 tags. iTRAQ labeled peptides were fractionated into 5 fractions and reconstituted, then separated in Dionex Ultimate NCR-3000RS (LC system, Sunnyvale, California, USA) and analyzed on an LTQ-Orbitrap Velos Pro (Thermo Scientific, Sunnyvale, California, USA). The iTRAQ MS/MS data was searched against UniProt human database (2015_12) using Proteome Discoverer 2.1 (Thermo Fisher Scientific). The abundance ratio data of sample to reference was log2 transformed before statistical analysis.

### Statistical analyses

Univariate statistical analyses were performed by Student’s test followed by correction for multiple testing according to Storey (q) [17]. Multivariate statistical modeling was performed using SIMCA 14.1 (MKS Umetrics, Umeå, Sweden) using principal component analysis (PCA), orthogonal projection to latent structure-discriminant analysis (OPLS-DA) [18] and partial least square (PLS) regression.

In contrast to the more commonly used PCA modeling, OPLS analysis is a supervised method designed to separate structured noise unrelated (orthogonal, often intra-group variance) to the predictive variance of interest (e.g., COPD patients vs healthy subjects). The resulting “noise filter” increases the interpretability of the multivariate model, particularly in deriving the observed group separation back to the specific proteins driving the separation. For more information of how to interpret these models, or the related model statistics, please see [19].

Proteins with a value of the scaled loadings of the first predictive component (|p(corr)[1]|) greater than the critical value of the Pearson correlation coefficient (*p* < 0.05) was considered significant for OPLS-DA models. Model performance is reported as the goodness of fit (R^2^), the goodness of prediction based on 7-fold cross validation (Q^2^), the cross-validated ANOVA (CV-ANOVA) [20] indicating the significance of group separation, and 200 times permutation test to determine the level of model statistics that may occur by random for the specific data set [21]. Multivariate correlation analysis between the specific proteins from identified pathways with clinical data was performed using PLS, and inner relations with Pearson correlation coefficient of *R*^2^ > 0.5 and *p* < 0.05 were considered significant.

### Pathway analysis

Pathway enrichment analysis was performed based on proteins found to be significantly altered in OPLS-DA models, as defined by OPLS-DA p(corr) above, using KOBAS 2.0 [22], with pathway enrichment analysis performed based on the KEGG pathway database [23].

## Results

### Clinical characteristics

FEV_1_ and percentage of neutrophils were lower (*p* < 0.05), while the proportion of macrophages was higher (*p* < 0.01) in Smokers compared to Never-smokers. No other differences between Smokers and Never-smokers, or between genders, were observed (Additional file 1: Table S1).

### Proteome alteration due to smoking

**Fig. 2:**
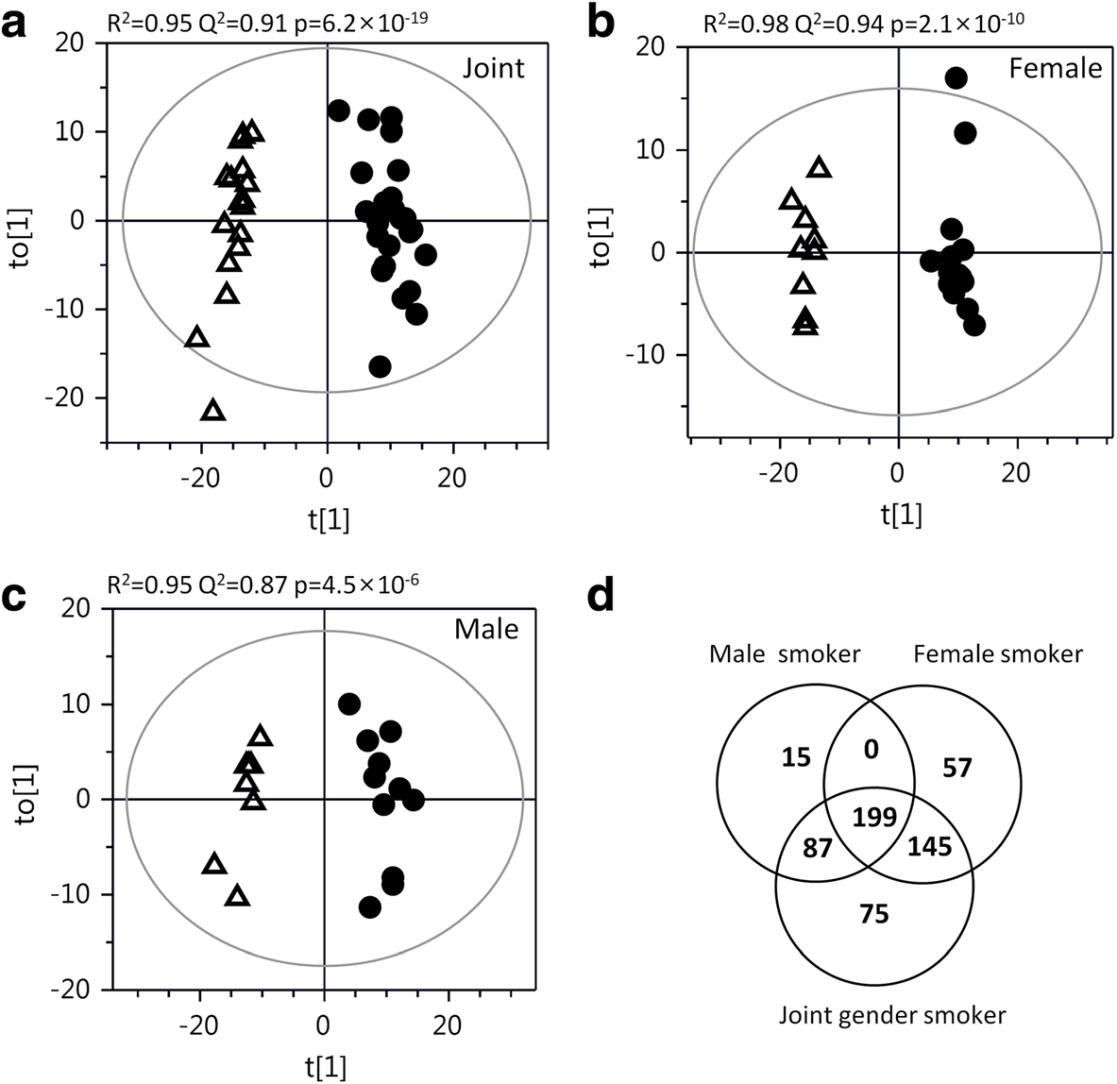
**a**) Scores plots of OPLS-DA modeling of BAL cell proteome alterations between Smokers (*circle*) vs Never-smokers (*open triangles*) OPLS-DA models suggested a perfect separation and predictive power between Smokers and Never-smokers p(CV-ANOVA) = 6.2 × 10^−19^) using 506 significant proteins (|p(corr)| > 0.34, *n* = 42, Additional file 1: Table S2). Stratification by gender revealed a highly significant separation in females (**b**; p(CV-ANOVA) = 3.2 × 10^−10^), fitted using 401 significant proteins (Additional file 1: Table S3), with a near perfect predictive power of 98% (*R*^2^ = 0.98, Q^2^ = 0.94). Also in males, the group separation was significant (**c**; p(CV-ANOVA) = 4.5 × 10^−6^), fitted with 301 significant proteins (Additional file 1: Table S4), with a predictive power of 87% (*R*^2^ = 0.95, Q^2^ = 0.87) . As displayed by the Venn diagram in panel **d**, a total of 199 proteins were altered in both female and male Smokers, while 202 proteins were altered only in female Smokers, and 102 proteins were altered only in male Smokers. An additional 75 proteins were found to be altered only in the joint gender model. The majority of the significantly altered proteins from the gender stratified models were altered also in the joint gender model; 95% in male and 86% female Smokers. Keys: t[1]: scores of OPLS-DA predictive component; to[1]: scores of the first orthogonal component from OPLS-DA model; p:cross-validated (CV)-ANOVA *p*-value for significance of group separation in the model

Univariate analysis comparing Never-smoker vs. Smoker groups revealed 610 significantly altered proteins (q < 0.05). Multivariate analysis using OPLS-DA showed a highly significant separation between the two groups (p[CV-ANOVA] = 6.2 × 10^−19^; Fig. 2a, Additional file 2: Figure S1) with 91% predictive power (*R*^2^ = 0.95, Q^2^ = 0.91), driven by 506 significant proteins (|p(corr)[1]| > 0.34, Additional file 1: Table S2).

To investigate the presence of any gender differences in response to smoking, statistical analyses were also performed following stratification by gender. Comparing female Smoker vs. Never-smoker groups, univariate statistical analysis showed 399 significantly altered proteins (q < 0.05). OPLS-DA models suggested that 401 proteins were significantly altered (|p[corr][1]| > 0.43, *n* = 23; Additional file 1: Table S3), driving a highly significant separation between groups (p[CV-ANOVA] = 2.1 × 10^−10^; Fig. 2b, Additional file 2: Figure S1) with a predictive power of 94% (*R*^2^ = 0.98, Q^2^ = 0.94,). For male Smoker vs. Never-smoker groups, univariate analysis identified 251 significantly altered proteins (q < 0.05). OPLS-DA modeling showed 301 significant proteins (|p(corr)[1]| > 0.50, *n* = 19; Additional file 1: Table S4), also here with significant separation between groups (p[CV-ANOVA] = 4.5 × 10^−6^; Fig. 2c; Additional file 2: Figure S1) and 87% predictive power (*R*^2^ = 0.95, Q^2^ = 0.87).

There was a considerable overlap of the proteins altered in males and females due to smoking (199 proteins, Fig. 2d). The majority of significant proteins in both gender smokers, 286 in male (95%) and 345 in female smokers (86%), were significantly altered also in the joint gender model.

### Alteration of pathway

**Fig. 3:**
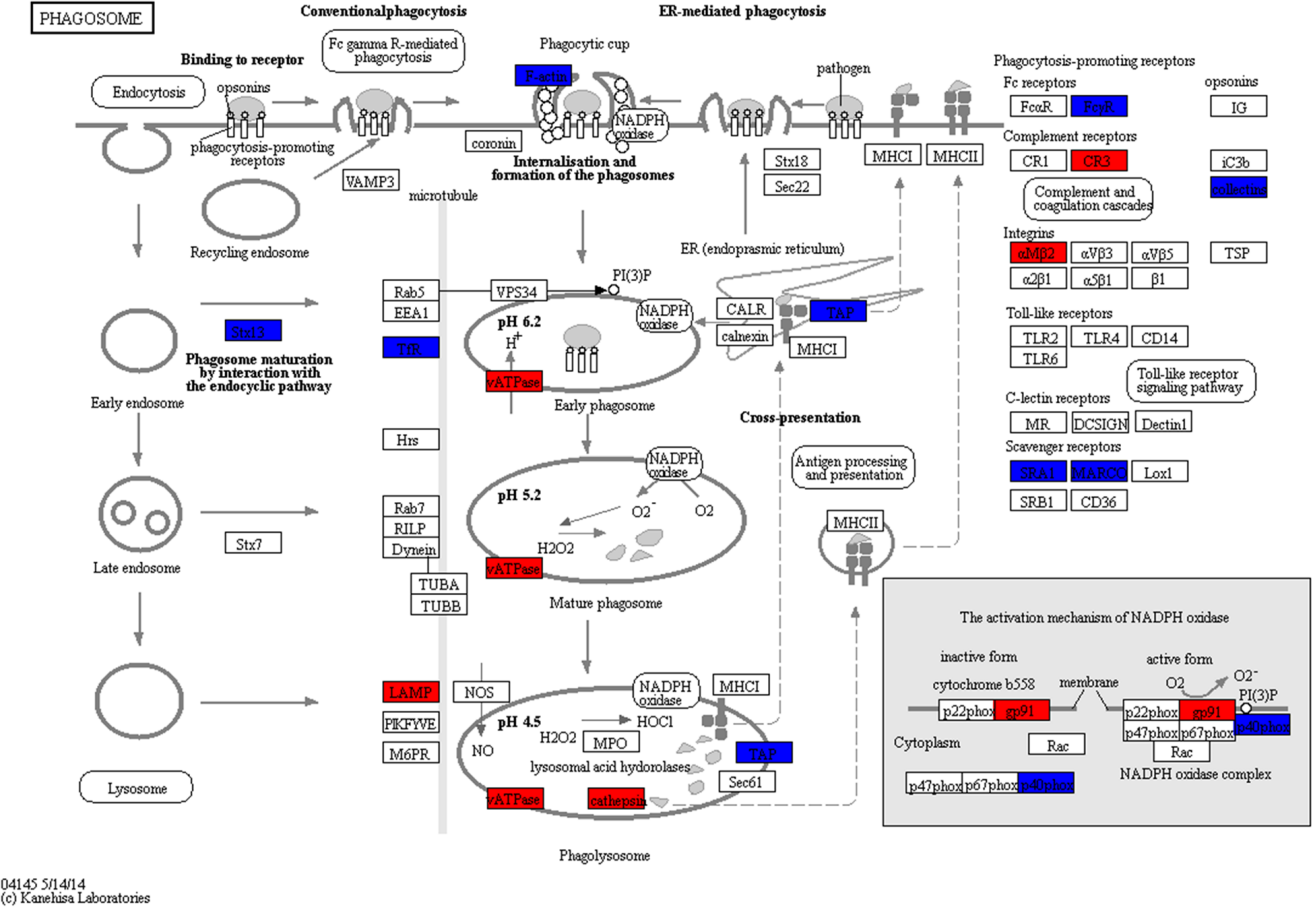
The phagosomal pathway was enriched in Smokers vs Never-smokers in the joint gender model (*p* = 1.4 × 10^−6^, FDR = 2.7 × 10^−5^). Proteins with significantly increased levels are highlighted in red, and decreased levels in dark blue. (FcγR, P08637, Low affinity immunoglobulin γ Fc region receptor III-A; CR3, P11215/P05107, CD11b/CD18; αMβ2, P11215/P05107, CD11b/CD18; Collectins, Q8IWL1, Pulmonary surfactant-associated protein A2; SRA1, P21757, Macrophage scavenger receptor types I and II; MARCO, Q9UEW3, Macrophage receptor MARCO; TAP1, Q03518, Antigen peptide transporter 1; TAP2, Q03519, Antigen peptide transporter 2; F-actin, P60709, β-actin; Stx13, Q86Y82, Syntaxin-12; TfR, P02786, Transferrin receptor protein 1; LAMP, P13473, lysosome-associated membrane glycoprotein 2; Cathepsin S: P25774, Cathepsin S; Cathepsin L1: P07711, Cathepsin L1; vATPase: P38606, P21281, P61421, P21283, Q9Y5K8, P36543, O75348, Q9UI12, V-ATPase subunit A, B2, d1, C1, D, E1, G1, H, respectively; gp91, P04839, Cytochrome b-245 heavy chain; p40phox, Q15080, Neutrophil cytosol factor 4. Protein details are listed in Additional file 1: Table S5)

Pathway analysis based on the 506 proteins found significant in the OPLS-DA model between Smokers and Never-smokers resulted in 15 significantly enriched pathways (pFDR < 0.05; Table 2, Additional file 1: Table S5). The majority of proteins in metabolic pathways such as oxidative phosphorylation, and citrate cycle were up-regulated, while ribosomal and antigen presentation were down-regulated in Smokers compared to Never-smokers. Proteins involved in the phagosomal (Fig. 3), LTM (Additional file 2: Figure S2) and lysosomal pathways were both up- and down-regulated (Table 2, Additional file 1: Table S5).

**Table 2:** Pathways analysis for joint gender, female, and male Smokers vs Never-smokers. Protein details are provided in Additional file 1: Tables S1, S2, S3 and S4

	Joint gender Smokers vs Never-smokers			Female Smokers vs Never-smokers			Male Smokers vs Never-smokers		
Pathway (No. of proteins in pathway)	Hits^a^	p-value	pFDR	Hits	p-value	pFDR	Hits	p-value	pFDR
Ribosome (180)	58(56↓)	3.4 × 10^−34^	7.1 × 10^−32^	53^b^(51↓)	1.2 × 10^−34^	2.6 × 10^−32^	37(36↓)	3.7 × 10^−26^	6.7 × 10^−24^
Oxidative phosphorylation (187)	32(31↑)	9.0 × 10^−13^	9.5 × 10^−11^	17(16↑)	2.1 × 10^−5^	6.3 × 10^−4^	22(21↑)	1.9 × 10^−11^	1.7 × 10^−9^
Lysosome (177)	29(22↑)	2.6 × 10^−11^	1.9 × 10^−9^	24(18↑)	4.4 × 10^−10^	4.7 × 10^−8^	20(13↑)	1.7 × 10^−9^	9.9 × 10^−8^
Citrate cycle (TCA cycle) (43)	15(15↑)	2.9 × 10^−10^	1.5 × 10^−8^	12(12↑)	1.1 × 10^−8^	8.1 × 10^−7^	11(11↑)	2.2 × 10^−8^	1.0 × 10^−8^
Valine, leucine and isoleucine degradation (64)	14(13↑)	1.7 × 10^−7^	6.0 × 10^−6^	11(11↑)	3.1 × 10^−6^	1.0 × 10^−4^	7(7↑)	6.0 × 10^−4^	6.0 × 10^−3^
Pyruvate metabolism (52)	12(10↑)	7.9 × 10^−7^	2.1 × 10^−5^	9(9↑)	2.3 × 10^−5^	6.0 × 10^−4^	8(7↑)	2.6 × 10^−5^	6.0 × 10^−4^
Phagosome (221)	24(13↑)	1.4 × 10^−6^	2.7 × 10^−5^	19(11↑)	1.5 × 10^−5^	5.0 × 10^−4^	17(12↑)	6.5 × 10^−6^	2.0 × 10^−4^
Fatty acid degradation (60)	10(8↑)	7.6 × 10^−5^	1.1 × 10^−3^	7(7↑)	1.6 × 10^−3^	0.026	6(6↑)	3.3 × 10^−3^	0.029
Glycolysis / Gluconeogenesis (101)	13(9↑)	7.7 × 10^−5^	1.1 × 10^−3^	11(11↑)	1.0 × 10^−4^	3.4 × 10^−3^	9(6↑)	2.0 × 10^−4^	2.8 × 10^−3^
Peroxisome (109)	13(10↑)	2.0 × 10^−3^	2.0 × 10^−3^	8(5↑)	0.010	0.01	8(6↑)	2.0 × 10^−3^	0.018
Fatty acid metabolism (62)	9(8↑)	4.0 × 10^−3^	5.4 × 10^−3^	8(8↑)	4 .0 × 10^−4^	8.0 × 10^−3^	6(6↑)	4.7 × 10^−3^	0.038
Propanoate metabolism (44)	7(5↑)	1.2 × 10^−3^	0.012	5(5↑)	8.0 × 10^−3^	0.10	2(1↑)	0.20	0.68
Fatty acid elongation	5(5↑)	4.9 × 10^−3^	0.04	5(5↑)	1.8 × 10^−3^	0.03	3(3↑)	0.02	0.14
Leukocyte transendothelial migration (166)	13(9↓)	5.3 × 10^−3^	0.045	8(6↓)	0.08	0.46	8(5↓)	0.018	0.13
Antigen presentation by MHC class I(28)^c^	6(5↓)	5.3 × 10^−5^	2.3 × 10^−3^	4(3↓)	2.1 × 10^−3^	0.045	4(3↓)	5.0 × 10^−4^	0.06

^a^Number of hits (number and direction of the majority of proteins). ^b^Number of proteins altered in female Smoker was higher than in males (χ^2^, *p* = 0.05); ^c^this pathway was analyzed using MetaCore

**Fig. 4:**
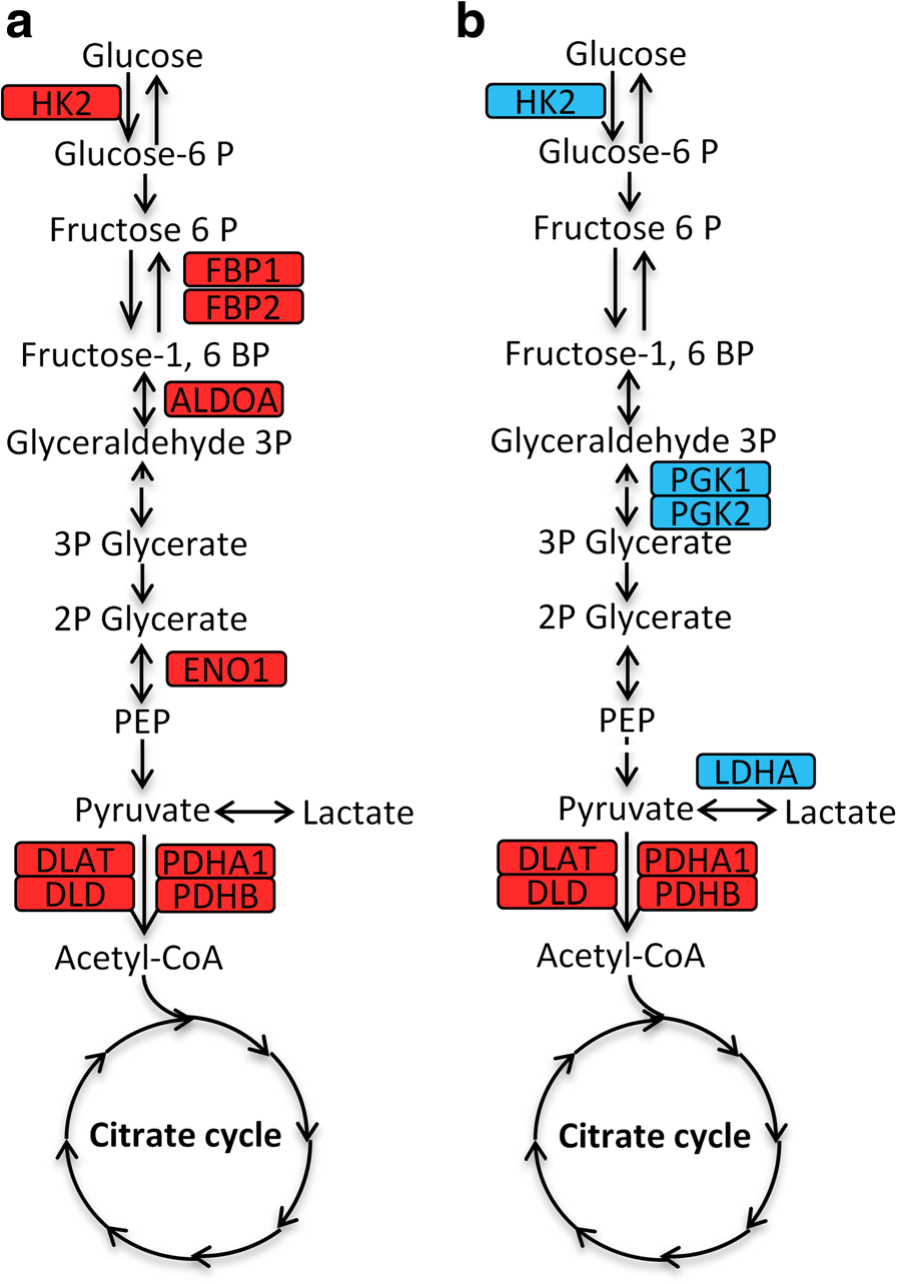
Gender-specific alterations in glycolysis/gluconeogenesis in Smokers. **a** The increased expression levels (*in red*) of five proteins (HK2, FBP1, FBP2, ALDOA and ENO1) in the female Smoker. **b** The decreased levels (*in blue*) of four proteins (HK2, PGK1, PGH2 and LDHA) in male Smokers. The levels of four proteins (DLAT, PDHA1, DLD and PDHB) in pathway pyruvate metabolism were increased in both gender Smokers

Gender-stratified pathway analysis showed that the majority of pathways mentioned above was altered in both genders in a similar fashion (Table 2, Additional file 1: Table S6), except the ribosomal- and glycolysis/gluconeogenesis pathways. In glycolysis/gluconeogenesis, five proteins were up-regulated in female Smokers (Fig. 4a), while four proteins were down-regulated in males (Fig. 4b).

**Fig. 5:**
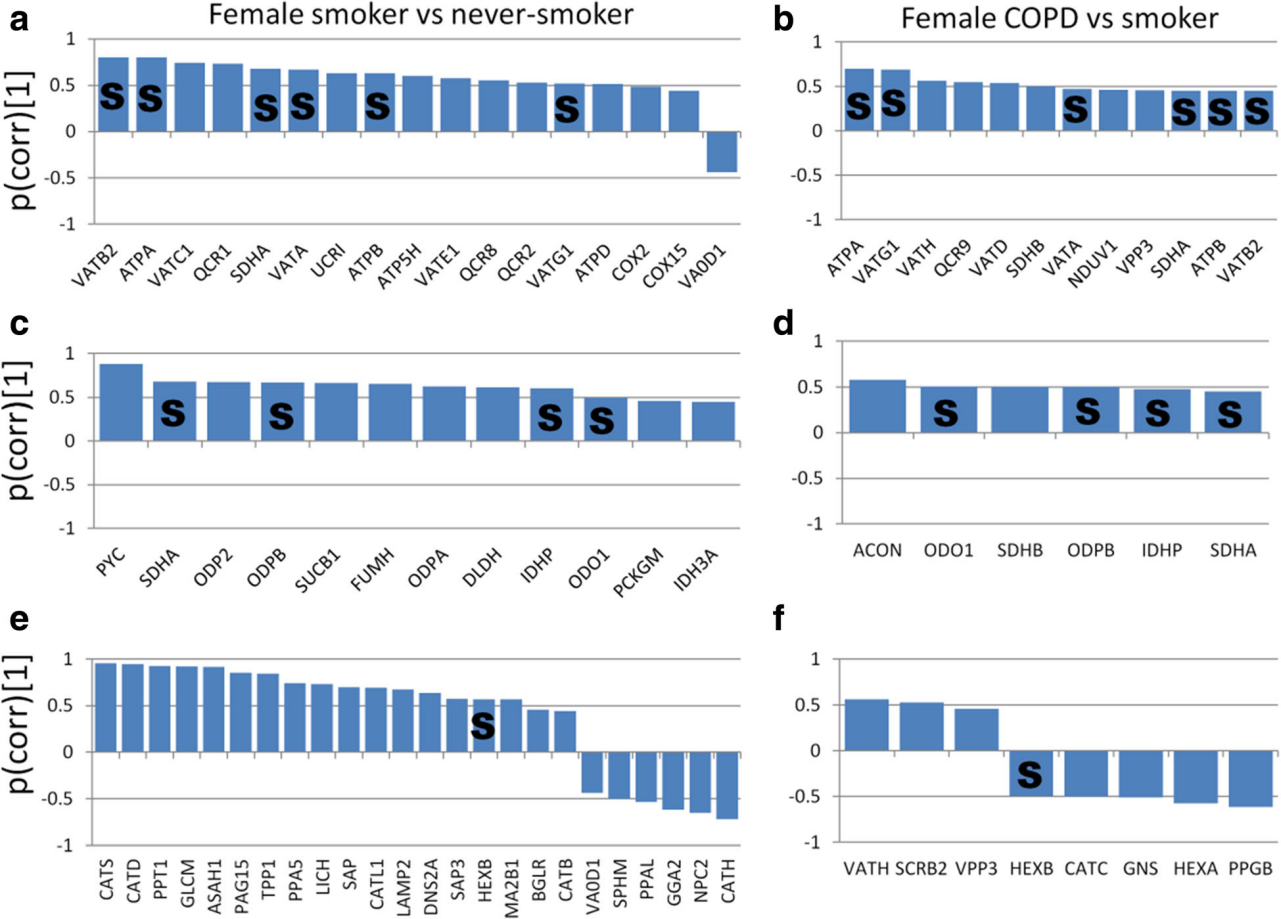
Pathways altered both due to smoking (left panels) and COPD (right panels) in females. The majority of expression levels in oxidative phosphorylation increased in the female Smoker compared to Never-smoker groups (**a**), and were further elevated in female current-smoker COPD compared with female Smoker groups (**b**), with six shared proteins (S). The levels of proteins in the citrate cycle were elevated in female Smokers compared to Never-smokers (**c**), and further increased in female smokers with COPD (**d**), with 4 proteins in common (S). The majority of protein levels in the lysosomal pathway were increased in female Smokers (**e**), but *decreased* in female smokers with COPD (**f**), with one common protein altered in the opposite direction (S). The bars display the scaled loadings, p(corr) of the predictive component from OPLS-DA models. Positive p(corr)[1] indicates increased protein levels. Full protein names are provided in Additional file 1: Table S3

Four pathways altered in Smokers were found to be altered in female Smokers with COPD: citrate cycle, oxidative phosphorylation, lysosome and phagosome [15]. The majority of protein levels in oxidative phosphorylation and citrate cycle increased in female Smokers (Fig. 5a, c) were further increased in COPD patients (Fig. 5b, d). For the lysosomal pathway, the majority of proteins increased in female Smokers, with the opposite trend in female smoking COPD patients, as indicated by the one shared protein (Fig. 5f).

### Phagosome and LTM pathways correlate with CD8^+^ T-cells and FEV_1_/FVC in smokers

**Fig. 6:**
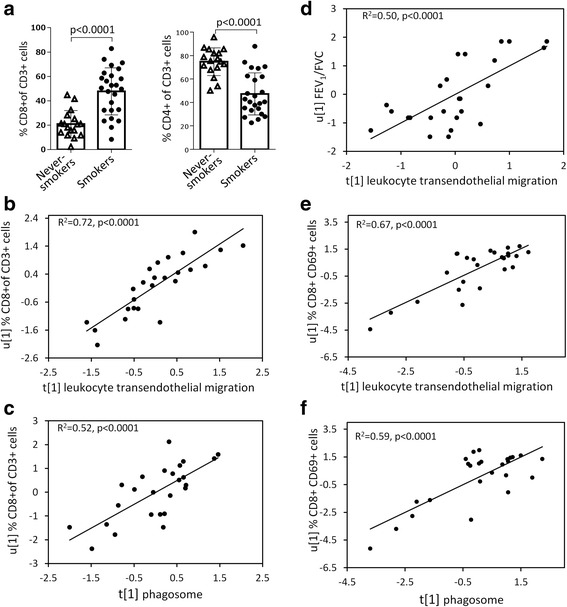
The proportion of CD8^+^ T cells in BAL increased and CD4^+^ T-cells decreased in Smokers compared to Never-smokers (**a**). Proteins from the leukocyte transendothelial migration (LTM) pathway and phagosome pathway correlated with proportion of CD8^+^ T-cells (**b-c**), as well as with the proportion of CD8^+^ CD69^+^ T-cells (**e-f**). Protein levels from the LTM pathway also correlated with the level of lung obstruction; FEV_1_/FVC (**d**). R^2^ and *p*-values refer to PLS inner relation

The proportion of CD8^+^ and CD4^+^ T-cells and several T-cell sub-phenotypes from BAL cells were quantified using flow cytometry [9, 11]. The proportion of CD8^+^ T-cells increased and CD4^+^ decreased (Fig. 6a, both *p* < 0.0001) in Smokers compared to Never-smokers. Accordingly, the ratio of CD4^+^ to CD8^+^ T-cells was lowered in Smokers as well (*p* < 0.0001). Correlation analyses revealed that proteins from the LTM- and phagosomal pathways significantly correlated with the proportion of CD8^+^ T-cells (*R*^2^ = 0.72, Fig. 6b; *R*^2^ = 0.52, Fig. 6c; both *p* < 0.0001, respectively, PLS inner relation) and FEV_1_/FVC (*R*^2^ = 0.50, Fig. 6d; *R*^2^ = 0.51, both *p* < 0.0001, respectively) in Smokers.

Several additional pathways showed correlations with CD8^+^ T-cells and FEV_1_/FVC. Further correlation analysis with CD8^+^ subpopulations showed that both the proteins of the LTM- and phagosomal pathways significantly correlated with the percentage of CD8^+^CD69^+^ cells (*R*^2^ = 0.67, Fig. 6e; *R*^2^ = 0.59, Fig. 6f; both *p* < 0.0001, respectively). No significant correlations were found with any of the other CD8^+^ sub-phenotype markers measured, including CD8^+^CXCR3, CD8^+^CCR5, CD8^+^CXCR4, CD8^+^CD103^+^.

### Proteomes identified by the iTRAQ vs. 2D–DIGE platforms

**Fig. 7:**
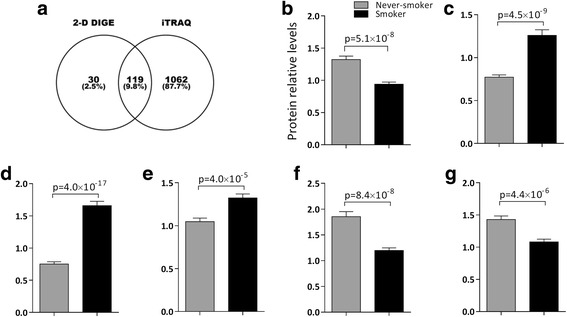
Comparison of the proteomes investigated with the bottom-up iTRAQ peptidomics platform and the top-down 2D–DIGE intact proteomics platform. **a**) Venn diagram displaying the overlap of proteins identified with the two platforms indicates that the majority of proteins identified with iTRAQ (90%) were novel as compared to 2D–DIGE. The proteins that overlapped were generally altered in a similar fashion, thereby providing validation across platforms. **b**) Protein 60S acidic ribosomal protein P0 with decreased level in ribosomal pathway in Smokers (2-D DIGE: *p* = 5.1 × 10^−8^; iTRAQ: *p* = 0.009); **c**) Protein Cytochrome b-c1 complex subunit Rieske with elevated level in pathway oxidative phosphorylation in Smokers (2-D DIGE: *p* = 4.5 × 10^−9^; iTRAQ: *p* = 1.5 × 10^−5^); **d**) Protein Cathepsin D with increased level in lysosomal pathway in Smokers (2D DIGE: *p* = 4.0 × 10^−17^; iTRAQ: *p* = 4.3 × 10^−19^); **e**) ATP synthase subunit d in mitochondria with upregulated level in citrate cycle in Smokers (2-D DIGE: p = 4.0 × 10^−5^; iTRAQ: *p* = 6.3 × 10^−5^); **f**) Enzyme Aldehyde dehydrogenase in mitochondria with downregulated level in pathway valine, leucine and isoleucine degradation in Smokers (2-D DIGE: *p* = 8.4 × 10^−8^; iTRAQ: *p* = 0.02); **g**) Protein actin, cytoplasmic 1 with decreased level in pathway phagosome in smokers (2-D DIGE: *p* = 4.4 × 10^−6^; iTRAQ: *p* = 0.007)

In complementary proteome investigations using 2D–DIGE on the same biospecimens from the same cohort, 149 proteins were identified [8]. Comparison of the proteomes investigated with the bottom-up iTRAQ peptidomics platform and the top-down 2D–DIGE intact proteomics platform indicates that the majority of proteins identified with iTRAQ (90%) were novel as compared to the previously published 2D–DIGE analyses Fig. 7a.

From the DIGE perspective, the majority of the proteins identified in the DIGE workflow were also identified using iTRAQ. However, it should be noted that there is a bias towards overlap since only proteins of interest are identified in the DIGE workflow. The proteins that overlapped were generally altered in a similar fashion, thereby providing validation across platforms. Some examples are given in Fig. 7b-g, showing the levels of six proteins from six pathways significantly altered in both platforms.

## Discussion

Here we used high-resolution iTRAQ based proteomics to investigate the BAL cell proteome of smokers with normal lung function (Smokers) vs. healthy never-smokers (Never-smokers), with the aim to explore alterations in specific proteins and molecular pathways occurring in response to long-term cigarette smoking in otherwise healthy individuals. The results clearly show that smoking exerted a considerable impact on the BAL cell proteome, with the abundances of half of the detected proteins being altered in long-term smokers. Downstream pathway enrichment analyses revealed that the 506 significantly altered proteins were associated with 15 molecular pathways (q < 0.05). Two of these pathways, the phagosome and leukocyte transendothelial migration, were significantly correlated with the proportion of CD8^+^ T-cell in BAL, as well as with the level of airway obstruction (FEV_1_/FVC) in the Smoker group. Some of the identified molecular pathways may represent molecular events preceding the development of smoking-related disease in susceptible individuals, while others may represent protective mechanisms in individuals that prove to be resilient to disease development in spite of decades of smoking.

The increase of CD8^+^ (cytotoxic) T-cells is one of the culprits of the inflammation and tissue destruction in the lung induced by long-term smoking in mice [24]. The level of lung cytotoxicity of CD8^+^ cells has been reported to increase with COPD severity [25], indicating that these cells were contributing to the pathogenesis of COPD. Increased levels of CD8^+^ T cells in lung parenchyma and pulmonary arteries have been reported as well [26]. In these studies, we observed an increase in the proportion of CD8^+^ T-cells in BAL cells of Smokers compared to Never-smokers, which is in line with our previous reports from the full Karolinska COSMIC cohort [9]. CD69 is a leukocyte activation marker, involved in the pathogenesis of chronic inflammation and activation of leukocyte subpopulations [27]. Recent studies have shown that CD69 is required for cigarette smoking-induced inflammation in the lung as well as the development of COPD [28, 29]. The significant correlations of proteins in the phagosome- and LTM pathways with activated cytotoxic T-cells (CD69+) and the level of obstruction (FEV_1_/FVC) in smokers, both hallmarks of COPD, suggest that dysregulation of these two pathways may play an important role in the molecular events preceding the development of COPD in susceptible smokers. The observations of further dysregulation in the phagosomal pathways in female smokers with mild to moderate COPD [15] further supports this theory.

The phagosome is formed by the fusion of cell membrane around large particles or pathogens [30], followed by invagination (phagocytosis). Nascent phagosomes interact with recycling endosomes, early endosomes and lysosomes, thereby acquiring digestive capacity and becoming phagolysosomes [31], where pathogens are killed and subsequently degraded into fragments. The levels of three phagocytotic proteins were altered in Smokers compared to Never-smokers: Syntaxin 12 and transferrin receptor 1, co-localize in the recycling endosome, with the function of facilitating the fusion of the early phagosome with early endosome [32]. LAMP2 is required for fusion of late phagosomes with lysosomes [33]. The dysregulation of these three proteins may indicated a disturbance in phagosome maturation due to smoking. Acidification is essential for the phagosomes to mature [34]. The observed increase in V-ATPases, which pump protons across the phagosomal membranes to maintain homeostasis of acidification, may indicate a dysregulation also in the local microenvironment of the phagosome. The decrease in F-actin and several receptors mediated in phagocytosis, including FcγR, SPA2, SRA1 and MARCO, may also contribute to defective phagocytosis in BAL immune cells due to smoking. Reduced protein levels of phagocytosis have been observed in female smokers with COPD [35]. Further dysregulation of the phagocytotic pathway in female smoking COPD patients compared to the Smokers studied here is reported in the companion paper [15]. As such, the level of dysregulation of the phagocytotic pathway in smokers may be of use as prognostic indicators of disease development.

The cell count of macrophages increased significantly in BAL fluid in Smokers. Alveolar macrophages predominantly originate from circulating blood monocytes [36], and are recruited to the lung by a mechanism involving leukocyte transendothelial migration (LTM), an inflammatory response to tissue damage. Recruitment of leukocytes from the blood to the site of injury includes several steps, including adhesion of leukocytes to the blood vessel wall, traversing the endothelial basement membrane, and migrating through the interstitial tissue [37]. Alterations of the LTM pathway were observed in the Smoker group, with a number of proteins being both up- and down-regulated.

Platelet/endothelial cell adhesion molecule 1 (PECAM-1, CD31), is expressed on platelets and leukocytes and concentrated at the borders of endothelial cells. PECAM-1 regulates the start of diapedesis and is required for transendothelial migration [38, 39]. The level of PECAM-1 decreased in BAL cells in Smokers, suggesting that LTM decreased in Smokers. PECAM-1 has previously been reported to be decreased in the serum of smoker compared with nonsmokers [40]. Myosin light-chain kinase (MLCK) and actin involve the actin-myosin fiber contraction, which helps endothelial cells separate [37]. The levels of these two molecules were reduced in BAL cells of smokers, further supporting that LTM may be reduced due to smoking.

Macrophage-1 antigen (Mac-1, CD11b, ITGAM) is expressed on the surface of many leukocytes including monocytes, neutrophils and macrophages. CD11b plays a key role in the regulation of leukocyte adhesion and migration in the inflammatory response [41], and in the recruitment of alveolar macrophage during e.g., pneumococcal infection [42]. Integrin beta-2 (CD18, ITGB2) pairs with CD11b to form the CR3 heterodimer of integrin. The higher expression levels of CD11b and CD18 indicated that, while the recruitment of leukocytes to the lung is impaired, leukocyte adhesion in the lungs increased due to smoking.

Four of the pathways (lysosome, citrate cycle, oxidative phosphorylation and phagosome) altered due to smoking were also found to be significantly altered in female smokers with COPD compared to smokers with normal spirometry, [8, 15]. The levels of the majority of proteins increased in the citrate cycle and oxidative phosphorylation pathways due to smoking were further increased in female smokers with COPD. The decreased levels of proteins in the lysosomal pathway observed in the female Smoker vs. Never-smoker groups were further decreased in female smoking COPD patients. As such, alteration of these pathways in smokers, with further enhanced dysregulation in smokers with mild to moderate COPD suggests that dysregulation of these pathways in healthy smokers may represent early signs of disease development in susceptible individuals. Even though the risk of developing COPD has been shown to increase linearly with the number of years smoked [43], it is curious that many smokers may never develop COPD in spite of decades of smoking. It is therefore plausible that some of the molecular pathways altered in long-term smokers represent protective mechanisms in resilient individuals, rather than disease mechanisms. These associations will be investigated following the completion of the on-going 10-year clinical follow-up of the Karolinska COSMIC cohort.

## Conclusion

Our high-resolution iTRAQ proteomics investigations of BAL cells from smokers with normal lung function and healthy never-smokers revealed that long-term smoking exerted a considerable impact on the BAL cell proteome, with almost half of the detected proteins representing 15 different molecular pathways being significantly altered. In contrast to the significant gender differences in proteome alterations observed between smokers with and without COPD diagnosis, the gender differences observed between Smokers and Never-smokers were minor. Proteins in the phagosome- and LTM pathways significantly correlated with the proportion of CD8^+^ T-cells, its subtype CD8^+^CD69^+^ cells as well as the level of airway obstruction (FEV_1_/FVC) in the Smoker group, stressing the importance these pathways may play both in the protection against as well as the early pathophysiology of smoking-induced inflammatory lung disorders such as COPD.

## Additional files

Additional file 1: **Table S1.** Clinical characteristics of subjects, stratified by gender. **Table S2.** Proteins significantly altered between Smoker vs Never-smoker groups. **Table S3.** Proteins significantly altered between female Smoker vs Never-smoker groups. **Table S4.** Proteins significantly altered between male Smoker vs Never-smoker groups. **Table S5.** Significantly enriched pathways and associated proteins when comparing Smoker and Never-smoker groups. **Table S6.** Significantly enriched pathways following stratification by gender when comparing Smoker and Never-smoker groups. (XLSX 1321 kb)

Additional file 2: **Figure S1.** The OPLS-DA modeling parameters for joint gender, female and male Smoker vs. Never-smoker. Permutation test was performed 200 times for each model. **Figure S2.** Leukocyte transendothelial migration was significantly altered in joint smokers. ITGAM, P11215, Integrin alpha-M (CD11b); ITGB2, P05107, Integrin beta-2 (CD18); PECAM1, P16284, Platelet endothelial cell adhesion molecule; JAM-A, Q9Y624, Junctional adhesion molecule A; MLC-2, O14950, Myosin regulatory light chain 12B; CDC42, P60953, Cell division control protein 42 homolog; Actin, P60709, actin cytoplasmic 1; α-actin, P12814, O43707, α-actinin-1, α-actinin-4, respectively; NOX2, P04839, Cytochrome b-245 heavy chain; p40phox, Q15080, Neutrophil cytosol factor 4; RAC2, P15153, Ras-related C3 botulinum toxin substrate 2; RAP1A, P62834, Ras-related protein Rap-1A. (DOC 1606 kb)

## Abbreviation

2D–DIGE: Two-dimensional difference gel electrophoresis
BAL: Bronchoalveolar lavage
COPD: Chronic obstructive pulmonary disease
FDR: False discovery rate
iTRAQ: Isobaric tags for relative and absolute quantitation
KEGG: Kyoto encyclopedia of genes and genomes
LTM: Leukocyte transendothelial migration
OPLS-DA: Orthogonal projection to latent structure-discriminant analysis
p(corr)[1]: Scaled loadings of the predictive component of OPLS-DA model
p(CV-ANOVA): *p*-value of cross-validated ANOVA
PCA: Principal component analysis
pFDR: Corrected p-value using Benjamini and Hochberg’s FDR method
PLS: Partial least square regression
Q^2^: Goodness of prediction (based on 7-fold cross-validation)
R^2^: Goodness of fit (determination coefficient)
SUS plot: The plot of shared and unique structure
