# Supplementary material for: Long-term smoking alters abundance of over half of the proteome in bronchoalveolar lavage cell in smokers with normal spirometry, with effects on molecular pathways associated with COPD
Source: Respir Res. 2018 Mar 8;19:40. doi: 10.1186/s12931-017-0695-6 (PMC5842534; doi:10.1186/s12931-017-0695-6)
Supplement: Supplementary file 2 — Figure S1. The OPLS-DA modeling parameters for joint gender, female and male Smoker vs. Never-smoker. Permutation test was performed 200 times for each model. Figure S2. Leukocyte transendothelial migration was significantly altered in joint smokers. ITGAM, P11215, Integrin alpha-M (CD11b); ITGB2, P05107, Integrin beta-2 (CD18); PECAM1, P16284, Platelet endothelial cell adhesion molecule; JAM-A, Q9Y624, Junctional adhesion molecule A; MLC-2, O14950, Myosin regulatory light chain 12B; CDC42, P60953, Cell division control protein 42 homolog; Actin, P60709, actin cytoplasmic 1; α-actin, P12814, O43707, α-actinin-1, α-actinin-4, respectively; NOX2, P04839, Cytochrome b-245 heavy chain; p40phox, Q15080, Neutrophil cytosol factor 4; RAC2, P15153, Ras-related C3 botulinum toxin substrate 2; RAP1A, P62834, Ras-related protein Rap-1A. (DOC 1606 kb) [file 12931_2017_695_MOESM2_ESM.doc]

**Long-term smoking alters abundance of over half of the proteome in bronchoalveolar lavage cell in smokers with normal spirometry, with effects on molecular pathways associated with COPD**

Mingxing Yang^1*^, M.D., Maxie Kohler^1^, Ph.D., Tina Heyder^1^, MSc, Helena Forsslund^1^, Ph.D., Hilde K. Garberg^2^, Ph.D., Reza Karimi^1^, M.D., Ph.D., Johan Grunewald^1^, M.D, Ph.D., Frode S. Berven^2^, Ph.D., C. Magnus Sköld^1^, M.D, Ph.D., Åsa M. Wheelock^1*^ Ph.D.

^1^Respiratory Medicine Unit, Department of Medicine Solna & Center for Molecular Medicine, Karolinska Institutet, Stockholm, Sweden; ^2^Proteomics Unit (PROBE), Department of Biomedicine, University of Bergen, Bergen, Norway

***Correspondence to be addressed to:**

Åsa M. Wheelock

Lung Research Lab L4:01, Respiratory Medicine Unit, Dept. of Medicine, Karolinska Institutet, SE-171 76, Stockholm, Sweden
Email: [asa.wheelock@ki.se](mailto:Maxie.kohler@ki.se) or

or

Mingxing Yang

Lung Research Lab L4:01, Respiratory Medicine Unit, Dept. of Medicine, Karolinska Institutet, SE-171 76, Stockholm, Sweden

Email: mingxing.yang@ki.se

**Running title:** Alterations in BAL cell proteome due to smoking

**Funding**

This study was funded by the Swedish Heart-Lung Foundation, Swedish Foundation for Strategic Research (SSF), VINNOVA (VINN-MER), EU FP6 Marie Curie, AFA Insurances, the King Oscar II Jubilee Foundation, the King Gustaf V and Queen Victoria’s Freemasons Foundation, the Swedish Research Council (VR), the regional agreement on medical training and clinical research (ALF) between Stockholm County Council and Karolinska Institutet.

The authors declared that they have no Conflict of interest.

## Methods

**Study Subjects and design**

This study was carried out on the subjects from the Karolinska COSMIC cohort (ClinicalTrials.gov identifier NCT02627872)[^1-6^](#_ENREF_1). The study was approved by the Stockholm regional ethical board (Case no. 2006/959-31/1) and the experiments conformed to the principles of the WMA Declaration of Helsinki, and written consent was obtained from all subjects. The Karolinska COSMIC study is a three group cross-sectional study in which each group was stratified by gender. The overall aim of the study is to investigate the differentiation between genders in response to smoking as well as early stage COPD by integrating several aspects of COPD and smoking through the use of imaging, transcriptomics, proteomics, metabolomics, and lymphocyte profiling in the context of clinical phenotypes. The study includes three age- (45-65 years) and gender matched groups of healthy never-smokers (Never-smokers), smokers with normal lung function (Smokers), and smokers with mild to moderate disease COPD (GOLD stage I-II, FEV1>50% and FEV_1_/FVC<0.7). In the study segment presented here, 42 individuals from the groups of Never-smokers (n=17) and Smokers (n=25) were included. Results from the Smokers compared to the COPD group are presented in the companion paper (Mingxing Yang, et al. companion manuscript). All subjects underwent extensive clinical examinations, including computed tomography and spirometry. Lung function parameters were calculated as previously described[^7^](#_ENREF_7). Smokers were defined as those with a smoking history of >10 pack years, and a current cigarette consumption of >10 cigarettes/day during the past 6 months. BAL cells were collected during bronchoscopy as previously reported[^8^](#_ENREF_8). Clinical characteristics are summarized in Table 1. There were no significant differences in age, height, weight, BMI, FEV_1_/FVC and BAL cell percentages s of lymphocytes, eosinophils, basophils and mast cells between Smokers and Never-smokers. FEV_1_ and percentage of neutrophils were lower (p<0.05), while the proportion of macrophages and was higher (p<0.01) in Smokers compared to Never-smokers. There were no differences in terms of smoking history (pack years), current cigarette consumption (cig/day during last 6 months), cell concentrations, FEV_1_ and FEV_1_/FVC between male and female Smokers (e-Table 1).

**Protein digestion and iTRAQ labeling**

Approximately 1.5×10^6^ BAL cells were pelleted and washed twice with PBS before lysis. Cell debris was removed by centrifugation and the supernatant was stored at -80ºC. Protein concentrations were determined using BCA protein assay kit (Thermo Fisher Scientific, Rockford, USA) according to the manufacturer’s description.

Reference samples were made by pooling 10 µg from all 69 protein samples (Never-smokers, Smokers and COPD patients) and divided into 20 µg aliquots and used as reference samples in all 23 iTRAQ experiments. All subjects protein samples and reference pool samples were reduced, cysteine blocked, trypsin digested, then labeled by 4-plexed iTRAQ according to the manufacturer’s instructions (AB SCIEX) with some modifications: Labeling was performed with half the amount of iTRAQ reagents, and the labeling reaction was increased to 1.5 hours. The reference sample in all experiments was labeled with the 114 tag, other samples were randomized and labeled with the 115, 116 and 117 tags.

**LC-MS/MS analyses and MS data preprocessing**

iTRAQ labeled peptides were fractionated into 5 fractions and reconstituted. Five mix mode fractions from each iTRAQ experiment were analyzed on an LTQ-Orbitrap Velos Pro (Thermo Scientific, Sunnyvale, California, USA) connected to a Dionex Ultimate NCR-3000RS (LC system, Sunnyvale, California, USA). The peptides were trapped on a pre-column in buffer A (2% ACN, 0.1% FA) at a flow rate of 5 µl/min for 5 minutes before separation by reverse phase chromatography at a flow of 280 nL/min. The mixed mode fractions were analyzed using two slightly different nano LC gradients. The first two fractions were run on a LC gradient starting at 5% buffer B (90% ACN, 0.1% FA) to finally to 90% B. The three last fractions were analyzed on a slightly steeper gradient, going from 25% B to 38% B. Full scan MS spectra were acquired in the Orbitrap with resolution R=120,000 at m/z 400. The 7 most intense eluting peptides above 1000 counts and charge states 2 or higher, were sequentially isolated in the linear ion trap. Fragmentation in the Higher-Energy Collision Dissociation cell was performed with a normalized collision energy of 40%, and activation time of 0.1 ms. Fragments were detected in the Orbitrap at a resolution of 15000.

The iTRAQ MS/MS data was searched against UniProt human database (2015_12) using Proteome Discoverer 2.1 (Thermo Fisher Scientific). Precursor mass tolerance was 10 ppm, product mass tolerance 0.5 Da and maximum one missed cleavages. Then the abundance ratio data of sample to reference was log2 transformed before statistical analysis.

**Statistical analyses**

Univariate statistical analyses were performed by Student’s test. Correction for multiple testing was performed according to Storey (q)[^9^](#_ENREF_9). Multivariate statistical modeling was performed using SIMCA 14.1 (MKS Umetrics, Umeå, Sweden) using principal component analysis (PCA), orthogonal projection to latent structure-discriminant analysis (OPLS-DA)[^10^](#_ENREF_10) and projection to latent structure (PLS). Significant variable in OPLS-DA modeling was defined by the scaled loadings, p(corr)[1]. Variables with |p(corr)[1]| ≥ the critical value of Pearson correlation coefficient corresponding to p<0.05 were considered significant and included in down-stream pathway analyses. Model optimization and variable selection were performed based on significance, as well as model statistics by optimizing model goodness-of-prediction (Q^2^) and significance of group separation. Model performance was estimated by the goodness of fit (R^2^), the goodness of prediction (Q^2^) and cross-validated ANOVA (CV-ANOVA)[^11^](#_ENREF_11) based on 7-fold cross-validation, and 200 times of permutation test, as previously described[^12^](#_ENREF_12). Multivariate correlation analysis between the specific proteins from identified pathways of interest with clinical data was performed using PLS, and inner relations with Pearson correlation coefficient of R^2^>0.5 (explained variance>50%) as well as p<0.05 were considered significant.

**Pathway analysis**

The lists of significant proteins, as defined by OPLS-DA p(corr) (see details above), were used to perform pathway enrichment analysis using KOBAS 2.0[^13^](#_ENREF_13), and the KEGG pathway database[^14^](#_ENREF_14).

References

1 Forsslund H, Mikko M, Karimi R, et al. Distribution of T-cell subsets in BAL fluid of patients with mild to moderate COPD depends on current smoking status and not airway obstruction. Chest 2014; 145:711-722

2 Kohler M, Sandberg A, Kjellqvist S, et al. Gender differences in the bronchoalveolar lavage cell proteome of patients with chronic obstructive pulmonary disease. J Allergy Clin Immunol 2013; 131:743-751

3 Karimi R, Tornling G, Forsslund H, et al. Lung density on high resolution computer tomography (HRCT) reflects degree of inflammation in smokers. Respir Res 2014; 15:23

4 Forsslund H, Yang M, Mikko M, et al. Gender differences in the T-cell profiles of the airways in COPD patients associated with clinical phenotypes. Int J Chron Obstruct Pulmon Dis 2017; 12:35-48

5 Balgoma D, Yang M, Sjodin M, et al. Linoleic acid-derived lipid mediators increase in a female-dominated subphenotype of COPD. Eur Respir J 2016; 47:1645-1656

6 Karimi R, Tornling G, Forsslund H, et al. Differences in regional air trapping in current smokers with normal spirometry. Eur Respir J 2017; 49:Accepted

7 Stocks J, Quanjer PH. Reference values for residual volume, functional residual capacity and total lung capacity. ATS Workshop on Lung Volume Measurements. Official Statement of The European Respiratory Society. Eur Respir J 1995; 8:492-506

8 Lofdahl JM, Cederlund K, Nathell L, et al. Bronchoalveolar lavage in COPD: fluid recovery correlates with the degree of emphysema. Eur Respir J 2005; 25:275-281

9 Storey JD. A direct approach to false discovery rates. Journal of the Royal Statistical Society: Series B (Statistical Methodology) 2002; 64:479-498

10 Bylesjö M, Rantalainen M, Cloarec O, et al. OPLS discriminant analysis: combining the strengths of PLS-DA and SIMCA classification. Journal of Chemometrics 2006; 20:341-351

11 Eriksson L, Trygg J, Wold S. CV-ANOVA for significance testing of PLS and OPLS® models. Journal of Chemometrics 2008; 22:594-600

12 Levanen B, Bhakta NR, Torregrosa Paredes P, et al. Altered microRNA profiles in bronchoalveolar lavage fluid exosomes in asthmatic patients. J Allergy Clin Immunol 2013; 131:894-903

13 Xie C, Mao X, Huang J, et al. KOBAS 2.0: a web server for annotation and identification of enriched pathways and diseases. Nucleic Acids Res 2011; 39:W316-322

14 Kanehisa M, Goto S, Sato Y, et al. KEGG for integration and interpretation of large-scale molecular data sets. Nucleic Acids Res 2012; 40:D109-114

# Supplemental Figures and Tables


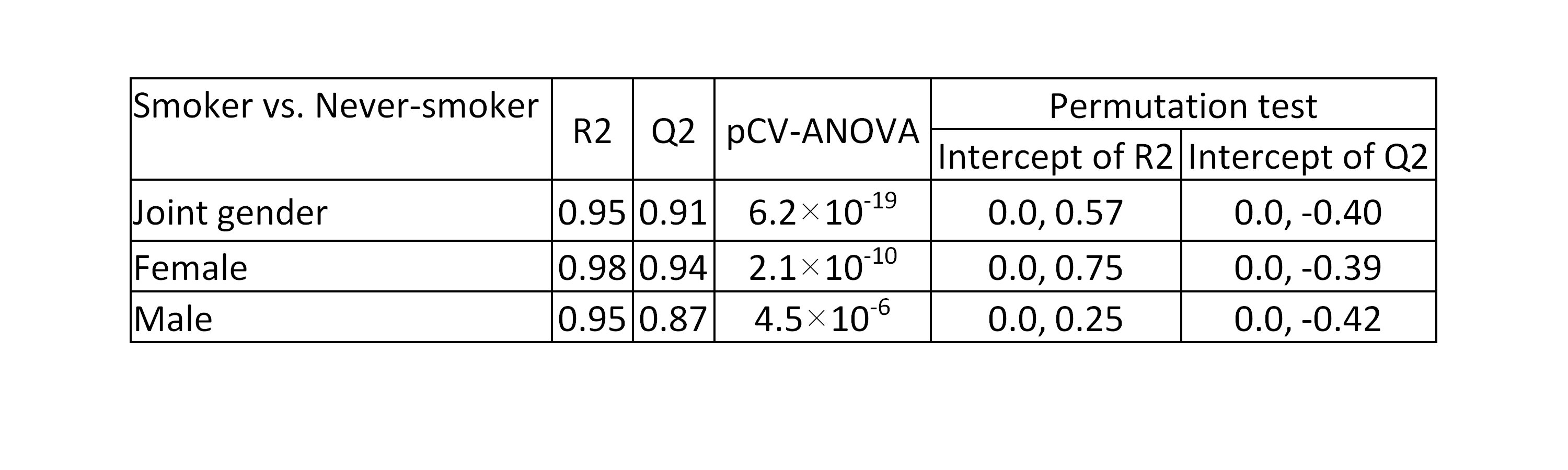


**e-Figure 1.** The OPLS-DA modeling parameters for joint gender, female and male Smoker vs. Never-smoker. Permutation test was performed 200 times for each model


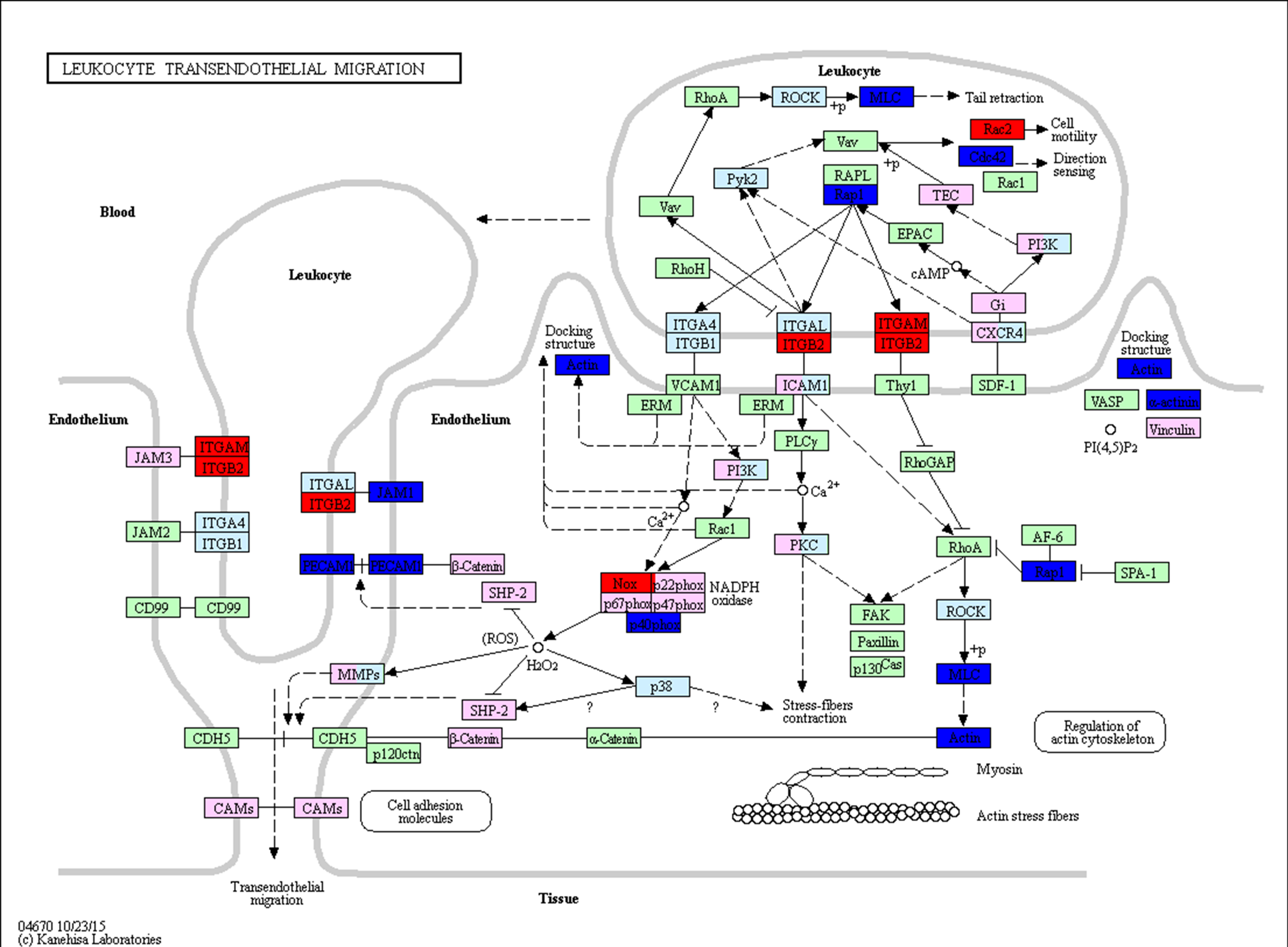


**e-Figure 2.** Leukocyte transendothelial migration was significantly altered in joint smokers. ITGAM, P11215, Integrin alpha-M (CD11b); ITGB2, P05107, Integrin beta-2 (CD18); PECAM1, P16284, Platelet endothelial cell adhesion molecule; JAM-A, Q9Y624, Junctional adhesion molecule A; MLC-2, O14950, Myosin regulatory light chain 12B; CDC42, P60953, Cell division control protein 42 homolog; Actin, P60709, actin cytoplasmic 1; α-actin, P12814, O43707, α-actinin-1, α-actinin-4, respectively; NOX2, P04839, Cytochrome b-245 heavy chain; p40phox, Q15080, Neutrophil cytosol factor 4; RAC2, P15153, Ras-related C3 botulinum toxin substrate 2; RAP1A, P62834, Ras-related protein Rap-1A.

**Supplemental Information e-Tables in a separate excel file**

e-Table 1. Clinical characteristics of subjects, stratified by gender

e-Table 2. Proteins significantly altered between Smoker vs Never-smoker groups

e-Table 3. Proteins significantly altered between female Smoker vs Never-smoker groups

e-Table 4. Proteins significantly altered between male Smoker vs Never-smoker groups

e-Table 5. Significantly enriched pathways and associated proteins when comparing Smoker and Never-smoker groups

e-Table 6. Significantly enriched pathways following stratification by gender when comparing Smoker and Never-smoker groups
